# Clinical characteristics of postoperative necrotizing enterocolitis in patients with congenital jejunoileal atresia and its risk factors

**DOI:** 10.1136/wjps-2023-000622

**Published:** 2024-03-18

**Authors:** Xiaofeng Xiong, Wei Lu, Fuzhong Xing, Yuan Cai, Jixin Yang, Yuhang Yuan, Jiexiong Feng, Xuyong Chen

**Affiliations:** 1 Department of Pediatric Surgery, Tongji Hospital of Tongji Medical College of Huazhong University of Science and Technology, Wuhan, Hubei, China; 2 Department of Neonate Surgery, Wuhan Children's Hospital, Huazhong University of Science and Technology, Wuhan, Hubei, China; 3 Department of Pediatric Surgery, The First Affiliated Hospital of Zhengzhou University, Zhengzhou, Henan, China

**Keywords:** Infectious Disease Medicine, Pediatrics, Congenital Abnormalities

## Abstract

**Objective:**

To review postoperative necrotizing enterocolitis (NEC) in patients with jejunoileal atresia (JIA) and to explore the potential risk factors related to the concurrence of NEC.

**Methods:**

Patients diagnosed with JIA who received surgical treatment from January 2016 to June 2021 were enrolled. Demographics, viral infection of the fetus, transfusion within 48 hours before NEC, sepsis before JIA repair, pathological and anatomical classification of JIA, combined malformation, occurrence time of NEC after the operation, treatment, and prognosis of patients were analyzed. Patients were divided into NEC group and non-NEC group, and all patients were followed up for 3–6 months to observe for complications.

**Results:**

A total of 180 patients with JIA were included, of whom 12 were diagnosed with NEC after surgery and 1 patient with NEC died during follow-up. The average age, birth weight, gestational age, proportion of premature infants, proportion of preoperative infections, and pathological classification of JIA did not significantly differ between the two groups. The probability of patients with proximal jejunal atresia (PJA) in the NEC group (58.3%) was higher than that in the non-NEC group (22.6%) (*p*=0.011), and patients with PJA had longer parenteral nutrition time than patients without PJA (26.64±9.21 days vs 15.11±6.58 days, *p*<0.001).

**Conclusion:**

PJA was more likely to be associated with concurrent NEC after surgery, which is a highly NEC-related risk factor inherent in JIA.

**WHAT IS ALREADY KNOWN ON THIS TOPIC**

- Jejunoileal atresia might have relation with the occurrence of necrotizing enterocolitis (NEC).

**WHAT THIS STUDY ADDS**

- Proximal jejunal atresia (PJA) was more likely to be associated with concurrent NEC after surgery.

**HOW THIS STUDY MIGHT AFFECT RESEARCH, PRACTICE OR POLICY**

- Full resection of the proximal lesioned duodenal segment could reduce the risk of NEC after PJA repair.

## Introduction

Jejunoileal atresia (JIA) is a common cause of intestinal obstruction in neonates, with an incidence of around 3 in 10 000 live births.^1^ Surgery is the predominant treatment for JIA, and even though most patients recover after an operation some patients experience concurrent complications, including enterodynamic disorders and bacterial translocation; necrotizing enterocolitis (NEC) is the rare but most serious complication.^2 3^ The risk factors for postoperative NEC include preterm birth, low body weight, low Apgar score, low gestational age, and red blood cell transfusion, but some unknown risk factors still exist.^4^ It is certain that NEC increases hospital stays and medical costs for patients with intestinal atresia after JIA repair. Identifying NEC-related risk factors of patients with JIA may help avoid these risks earlier and provide timely treatment. In this study, we intended to report the clinical characteristics, management, and prognosis of patients with NEC after JIA repair and determine its related risk factors.

## Methods

### Patients

Patients diagnosed with JIA who underwent surgery at the Department of Neonatal Surgery of Wuhan Children’s Hospital from January 2016 to June 2021 were enrolled. The exclusion criteria were as follows: patients with a diagnosis of JIA complicated with malformations, including meconium peritonitis, malrotation of the intestine, omphalocele, or annular pancreas; birth with asphyxia; complication with intrauterine infection or thrombocytopaenia (less than 100×10^9^/L); complication with severe congenital heart disease (such as tetralogy of Fallot or double outlet of the right ventricle); and maternal complications during pregnancy. Patients were divided into NEC group and non-NEC group. The clinical data of the patients between the two groups were compared. The anatomical classification, pathological classification, parenteral nutrition duration, and risk assessment of NEC after JIA repair were assessed.

### Data collection and definition

Clinical data including age, sex, premature birth (<37 weeks’ gestational age), birth weight, mode of delivery, intrauterine infection, blood transfusion within 48 hours before NEC, patent ductus arteriosus (PDA), patent foramen ovale, preoperative sepsis, pathological and anatomical classification of intestinal atresia, postoperative feeding, duration of NEC recurrence after the operation, treatment method, and prognosis were collected. Laboratory examinations including routine blood analysis, C reactive protein analysis, X-ray, and abdominal CT were performed when abdominal distension, bloody stools, and infection indicators appeared.

The diagnostic criteria for postoperative NEC were as follows: Bell stage II or above with at least one or more gastrointestinal symptoms, such as bilious emesis or gastric aspirate; blood in the stool without a rectal fissure; abdominal distension; and at least one radiographic sign, such as pneumatosis intestinalis, pneumoperitoneum, or hepatobiliary gas.^5^ Patients suspected of having NEC were excluded.

Proximal jejunal atresia (PJA) was defined as jejunal atresia within 10 cm of the Treitz ligament, while non-PJA referred to atresia located 10 cm away from the Treitz ligament. Pathological classification of JIA includes type I, type II, type IIIA, type IIIB, and type IV, as previously reported.^2 6^

### Surgical management

For the treatment of PJA, we removed the dilated segments of the duodenum and proximal jejunum as much as possible and performed jejunoduodenostomy. The first and second segments were preserved due to the anatomical structure of the large duodenal papilla. During hospitalization, patients were fed with hydrolyzed protein milk.

### Statistical analysis

All analyses were performed with SPSS V.18.0 software. Independent sample t-test was used for comparison of quantitative variables between groups and χ^2^ was used for comparison of qualitative variables. *P*<0.05 was considered statistically significant.

## Results

### Clinical characteristics of patients with NEC after JIA repair

Among 180 included patients, 12 (6.7%) were diagnosed with NEC after JIA repair. The male to female ratio was 3:1, the average birth weight was 2.67±0.65 kg, and the average gestational age was 36.7 weeks. Five patients (41.7%) were found to have PDA and 10 (83.3%) patients were found to have atrial septal defect (ASD). The average total bilirubin level was 106.61±65.6 µmol/L. There were two patients with white cell count greater than 10×10^9^/L. The average amount of milk consumed when NEC occurred was 9.01±3.51 mL/kg each time, and the average postoperative days when NEC occurred was 21.5±5.90 days. The overall clinical characteristics of patients with NEC after JIA repair are summarized in table 1 and are similar to that of patients with sporadic NEC.

**Table 1:** Basic clinical data of patients with NEC after JIA repair

Case	1	2	3	4	5	6	7	8	9	10	11	12
Gender	M	M	F	M	M	M	M	F	M	M	F	M
Age (days)	1	2	3	3	2	2	1	3	1	3	2	3
Weight (kg)	2.41	3.8	2	2.2	2.45	3.5	2.5	2.25	3.5	3.15	1.87	2.4
Pregnancy (weeks)	37	40	36	36	35	38	37	34	41	37	32	37
Pathological classification	II	IIIB	IIIA	IV	IIIB	IIIB	IV	I	IIIA	II	IIIA	IIIA
Anatomical classification	Non-PJA	PJA	Non-PJA	PJA	PJA	PJA	PJA	PJA	Non-PJA	PJA	Non-PJA	Non -PJA
Congenital heart disease	PDA	PDA	PDA	–	–	PDA	–	–	–	–	–	PDA
	ASD	ASD	ASD	ASD	ASD	ASD	–	ASD	ASD	ASD	ASD	–
Total bilirubin (μmol/L)	251.1	69.1	82.5	223.7	38.92	112.1	117.9	56	78	102	90	58
White cell count (×10^9^/L)	11.4	5.55	6.18	6.5	4.2	5.58	0.82	7.33	5.1	34.2	5.84	3.29
Milk consumed when NEC occurs (mL/kg)	10.37	5.26	12.5	15.91	10.2	11.43	10	8.89	5.71	3.17	8.02	6.67
Postoperative days when NEC occurs (days)	16	18	21	26	14	15	21	35	27	24	20	21

ASD, atrial septal defect; F, female; JIA, jejunoileal atresia; M, male; NEC, neonatal necrotizing enterocolitis; PDA, patent ductus arteriosus; PJA, proximal jejunal atresia.

### Comparisons between patients with and without NEC after JIA repair

The average age, weight, total bilirubin concentration, premature liver ratio, infection ratio, proportion of combined PDA and ASD, and pathological classification were not significantly different between the two groups (table 2). We found significantly higher proportion of patients with PJA in the NEC group (58.3%) than in the non-NEC group (22.6%) (*p*=0.011) (table 2). We also found that patients with PJA had longer parenteral nutrition time than patients without PJA (26.64±9.21 days vs 15.11±6.58 days, *p*<0.001) in all patients, while parenteral nutrition time was not significantly different between patients with PJA and those without PJA in the NEC group (35.43±8.90 days vs 27.2±6.72 days, *p*=0.108). Eleven patients with NEC recovered from medical treatment without complications, and one patient with NEC who also had extensive intestinal necrosis died.

**Table 2:** Comparisons of clinical, pathological, and anatomical results between patients with and without NEC after JIA repair

Variables	Patients with NEC (*n*=12)	Patients without NEC (*n*=168)	t/χ^2^	*P* value
Age (days)	2.17±0.83	1.65±0.70	2.115	0.056*
Weight (kg)	2.67±0.65	3.04±0.55	−1.93	0.077*
Total bilirubin (μmol/mL)	106.61±65.6	147.74±85.1	−2.052	0.06*
Premature birth, *n* (%)	5 (41.7)	35 (20.8)	2.813	0.142†
Infection, *n* (%)	3 (25)	19 (11.3)	1.957	0.168†
PDA, *n* (%)	5 (41.7)	48 (28.6)	0.925	0.514†
ASD, *n* (%)	10 (83.3)	99 (58.9)	2.793	0.129†
Pathological classification, *n* (%)				
I	1 (8.3)	23 (13.7)	4.287	0.369†
II	2 (16.7)	42 (25.0)		
IIIA	4 (33.3)	72 (42.9)		
IIIB	3 (25.0)	22 (13.1)		
IV	2 (16.7)	9 (5.4)		
Anatomical classification, *n* (%)				
PJA	7 (58.3)	38 (22.6)	7.619	0.011†
Non-PJA	5 (41.7)	130 (77.4)		

Data are presented as mean±SD or number (percentage).

*t-test.

†χ^2^ test.

ASD, atrial septal defect; JIA, jejunoileal atresia; NEC, necrotizing enterocolitis; PDA, patent ductus arteriosus; PJA, proximal jejunal atresia.

## Discussion

JIA is a common congenital intestinal disease in neonates. Surgical resection of the lesioned bowel is the major treatment method, but postoperative complications still affect patients’ lives. NEC, one of the most common life-threatening gastrointestinal diseases that affect premature and low birthweight infants, can also be detected after JIA repair. In the present study, we reported the demographic and pathological features of patients with or without NEC after JIA repair and found more patients with PJA in the NEC group than in the non-NEC group. Patients with PJA had longer parenteral nutrition time than patients without PJA.

PJA usually leads to overall expansion of the duodenum or even the giant duodenum; thus, intraoperative duodenectomy, clipping, and duodenal jejunal anastomosis are the major surgical procedures.^7^ However, it is still difficult to completely remove all the dilated duodenum, as the duodenal papilla should be intact. Moreover, pathological changes in the enteric nervous system and interstitial cells of Cajal in the residual region around the site of the intestinal atresia are the major factors that lead to the dysregulation of intestinal peristalsis and postoperative intestinal motility disorders.^8–11^ The above factors, combined with the reduced gradient pressure at the anastomotic site, ultimately result in poor passage of intestinal contents or even in the occurrence of NEC.^12^ In the present study, we observed that the incidence of NEC after JIA repair was not correlated with demographic or pathological features of the patients. We found that PJA was more likely to be concurrent with NEC. These findings suggest that anatomy is a key risk factor affecting the outcome of JIA and that surgeons should pay more attention to the anatomical location when these procedures are performed. Previous studies reported that the presence of micro-organisms in the immature bowel, the presence of food in the bowel, and triggering events that disrupt the integrity of the mucosal barrier are the three main factors for NEC development.^13 14^ In addition, the surgical procedure for intestinal atresia might be complicated by bacterial translocation, which is considered to be an important risk factor for NEC^13 15^; thus, additional evidence needs to be obtained to determine the risk factors for NEC occurrence after JIA repair.

The occurrence of NEC is not only related to susceptibility risk factors, but can also be related to the treatment, including inadequate human breast feeding, transfusion management, and feeding intolerance.^5^ A lack of enteral feeding can affect intestinal barrier function and reduce epithelial connections, which promote the translocation of bacteria and ultimately lead to intestinal inflammation.^16 17^ For patients with intestinal atresia, parenteral nutrition is essential for promoting intestinal recovery, and PJA may lead to proximal intestinal dysmotility, which affects the intestinal microenvironment, similar to microbial components. Changes in gut dysmotility and bacteria are correlated with the occurrence of NEC.^18 19^ Moreover, parenteral nutrition can also change gut microbiota dysbiosis, and long-term parenteral nutrition may predispose patients to Toll-like receptor 4 dependent NEC lesions.^20 21^ In our study, we found that the duration of parenteral nutrition in patients with PJA was longer than in patients without PJA, and patients with PJA were more likely to experience NEC, which indicate that the longer duration of parenteral nutrition in patients with PJA may be related to the occurrence of NEC in patients after JIA repair. The clinical features of patients with acute food protein-induced enterocolitis syndrome (FPIES) are similar to those with NEC, making differential diagnosis difficult, while the possibility of FPIES should be considered when clinical signs are not commensurate with symptoms of infection poisoning, abdominal signs, or infection indicators.^22^ In our cases, to avoid lactose intolerance and milk curds, we routinely administered hydrolyzed protein milk, which is a hypoallergenic formula, to avoid the occurrence of FPIES.

After the operation, the intestine needs a period of rest and rejuvenation to function optimally. In our study, the feeding quantity was 9.01±3.51 mL/kg/time when NEC occurred, which accounts for 60% of the total feeding (15 mL/kg/time). For neonates with gastrointestinal dysfunction, how to balance fasting and feeding, and avoid intestinal mucosal damage, intestinal barrier dysfunction, and bacterial translocation, and how to reduce the occurrence of postoperative NEC need to be further investigated.

In conclusion, we found that there was no correlation between the pathological classification of JIA and NEC occurrence, but PJA was more likely to occur in patients with postoperative NEC. Dilation of the duodenum is the anatomical basis for postoperative enterodynamic disorders. Balancing resection of the proximal lesioned duodenal segment with postoperative fasting–feeding will be a challenge for surgeons, and more attention needs to be paid to reduce the risk of NEC after JIA repair. One limitation of this study is that the sample size of the NEC group is small, and additional cases are needed to confirm the above conclusions.

## Data Availability

All data relevant to the study are included in the article or uploaded as supplementary information.
